# Association of baseline electrocardiographic left ventricular hypertrophy with future renal function decline in the general population

**DOI:** 10.1038/s41598-023-51085-1

**Published:** 2024-01-03

**Authors:** Shota Ikeda, Keisuke Shinohara, Koshiro Tagawa, Takeshi Tohyama, Junji Kishimoto, Masaya Kazurayama, Shinji Tanaka, Masamitsu Yamaizumi, Hirokazu Nagayoshi, Kensuke Toyama, Shouji Matsushima, Hiroyuki Tsutsui, Shintaro Kinugawa

**Affiliations:** 1 Department of Cardiovascular Medicine, Faculty of Medical Sciences, Kyushu University, 3-1-1 Maidashi, Higashi-ku, Fukuoka, 812-8582 Japan; 2 Center for Clinical and Translational Research, Kyushu University Hospital, Fukuoka, Japan; 3 JA Ehime Kouseiren Checkup Center, Ehime, Japan; 4 Department of Pharmacology, Ehime University Graduate School of Medicine, Ehime, Japan

**Keywords:** Disease prevention, Chronic kidney disease, Cardiology

## Abstract

Electrocardiographic left ventricular hypertrophy (LVH) could predict adverse renal outcomes in patients with hypertension. This study aimed to investigate the association between electrocardiographic LVH and future decline in renal function in the general population using a dataset of population-based health checkups from 2010 to 2019 including 19,825 participants. Electrocardiographic LVH was defined according to the Minnesota code. Renal function decline was defined as a decrease of ≥ 25% in the estimated glomerular filtration rate from baseline to < 60 mL/min/1.73 m^2^. Electrocardiographic LVH was found in 1263 participants at the baseline visit. The mean follow-up period was 3.4 ± 1.9 years. The incidence rates of renal function decline were 0.30 and 0.78 per 100 person-years in the non-LVH group and LVH groups, respectively. Electrocardiographic LVH was associated with the risk for renal function decline in the adjusted analysis (hazard ratio 1.69, 95% confidence interval 1.14–2.50, P = 0.009). This association was comparable across subgroups stratified by age, sex, body mass index, diagnosed hypertension, systolic blood pressure, hemoglobin A1c, and urinary protein. This study underscores the usefulness of electrocardiographic LVH to detect high-risk individuals for renal function decline in the setting of health checkups in the general population.

## Introduction

Chronic kidney disease (CKD) is recognized as a global health problem^1^. Approximately 10% of the American, European, and Asian populations are diagnosed with CKD^2^. In 2017, CKD was a cause of death in 1.2 million people worldwide, and the number of deaths attributable to end-stage kidney disease is expected to increase^3,4^. CKD is also associated with cardiovascular diseases as well as end-stage kidney disease^5,6^. Thus, early diagnosis and early intervention for CKD are important to reduce the risk of these events^7,8^.

Cardiovascular diseases and CKD are linked to each other, which is known as cardiorenal syndrome^9^. Congestive heart failure is a well-known risk factor for acute and chronic progression of kidney disorder. Left ventricular hypertrophy (LVH), a common cardiac abnormality, as well as heart failure might be associated with impaired renal function^10,11^. A population-based autopsy study showed the association between reduced estimated glomerular filtration rate (eGFR) and cardiac hypertrophy and fibrosis^12^. While electrocardiographic LVH is commonly observed in patients with hypertension, even mild renal dysfunction was associated with the presence of LVH on electrocardiogram^13^. Electrocardiographic LVH could predict the progression of CKD to a more advanced stage or to dialysis in patients with hypertension^14^. However, such an association between electrocardiographic LVH and future renal function decline has not been investigated in the general population.

The Japanese government launched an annual specific health checkup to detect noncommunicable diseases such as diabetes mellitus, hypertension, dyslipidemia, and CKD earlier, targeting all individuals aged 40–74 years. Individuals aged ≤ 39 years employed by a company are also obliged to receive health checkups annually. Despite the attempts to detect noncommunicable diseases, recognition of CKD at the early phase appeared to be suboptimal^15^. Hence, it is crucial to develop strategies for early detection of high-risk populations for future renal function decline, particularly among individuals who have not yet progressed to CKD stage G2, in the setting of health checkups. This study aimed to elucidate the association between electrocardiographic LVH and future renal function decline using data obtained from annual health checkups. Based on this study rationale, individuals with baseline eGFR < 60 mL/min/1.73 m^2^ were excluded, and the renal outcome was defined as a ≥ 25% decrease in eGFR from baseline to < 60 mL/min/1.73 m^2^, a less robust surrogate outcome, to identify individuals at high risk for CKD early in this study.

## Results

### Summary of the study and participants’ backgrounds

**Figure 1:**
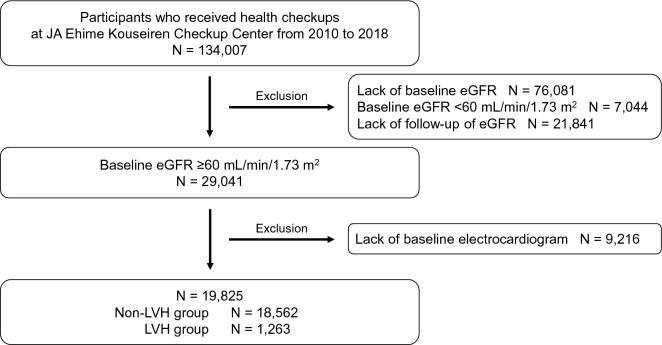
Flow diagram of participant selection. The study flow diagram and the number of individuals with or without electrocardiographic LVH. *eGFR* estimated glomerular filtration rate, *LVH* left ventricular hypertrophy.

A total of 134,007 individuals received health checkups at the JA Ehime Kouseiren Checkup Center at least once between 2010 and 2018 (Fig. 1). Individuals without baseline eGFR data (n = 76,081), without follow-up eGFR data (n = 21,841), and without baseline electrocardiography data (n = 9216) were excluded. Individuals with baseline eGFR < 60 mL/min/1.73 m^2^ (n = 7044) were also excluded in this study because individuals with eGFR < 60 mL/min/1.73 m^2^ might have CKD at baseline. Of the 19,825 individuals included in this study, 1263 had electrocardiographic LVH at the baseline visit, and 18,562 did not. The primary outcome was observed in 34 participants with electrocardiographic LVH and 189 participants without electrocardiographic LVH during a mean follow-up period of 3.4 ± 1.9 (median, 3.0; interquartile range, 1.8–5.0) years.

Table 1 shows the baseline characteristics of the participants. The LVH group was significantly older than the non-LVH group (57.3 ± 13.1 vs 53.6 ± 13.2 years, P < 0.001). Male sex (72.8 vs 46.3%, P < 0.001), current smoking (23.4 vs 19.1%, P < 0.001), and history of cardiovascular disease (7.8 vs 5.6%, P = 0.002) were more frequently observed in the LVH group than in the non-LVH group. Systolic blood pressure (BP) (136.7 ± 20.9 vs 123.7 ± 19.1 mmHg, P < 0.001) was significantly higher in the LVH group than in the non-LVH group. The complication rate of hypertension, which had already been diagnosed, was higher in the LVH group than in the non-LVH group (29.6 vs 17.9%, P < 0.001), whereas the prevalence of diabetes mellitus and dyslipidemia were comparable between the two groups. The values of eGFR, hemoglobin A1c (HbA1c), and low-density lipoprotein cholesterol (LDL-C) were comparable between the two groups, whereas the uric acid level (5.7 ± 1.4 vs 5.2 ± 1.4 mg/dL, P < 0.001) was higher in the LVH group than in the non-LVH group.

**Table 1:** Baseline characteristics stratified by the presence or absence of electrocardiographic left ventricular hypertrophy.

	Non-LVH group	LVH group	P value
	n = 18,562	n = 1263	
Age, years	53.6 ± 13.2	57.3 ± 13.1	< 0.001
Male, n (%)	8589 (46.3)	919 (72.8)	< 0.001
BMI, kg/m^2^	23.1 ± 3.7	23.1 ± 3.0	0.95
Systolic BP, mmHg	123.7 ± 19.1	136.7 ± 20.9	< 0.001
Current smoking, n (%)	3537 (19.1)	293 (23.4)	< 0.001
History of CVD, n (%)	1034 (5.6)	98 (7.8)	0.002
Hypertension, n (%)*	3329 (17.9)	374 (29.6)	< 0.001
Diabetes mellitus, n (%)	1031 (5.6)	78 (6.2)	0.35
Dyslipidemia, n (%)	2562 (13.8)	160 (12.7)	0.26
eGFR, mL/min/1.73 m^2^	80.9 ± 13.9	80.9 ± 14.3	0.90
Urinary protein positive, n (%)	581 (3.1)	71 (5.6)	< 0.001
HbA1c, %	5.6 ± 0.6	5.6 ± 0.6	0.77
LDL-C, mg/dL	124.9 ± 32.5	125.6 ± 30.9	0.46
Uric acid, mg/dL	5.2 ± 1.4	5.7 ± 1.4	< 0.001

*Prior diagnosis of hypertension.

*BMI* body mass index, *BP* blood pressure, *CVD* cardiovascular diseases, *eGFR* estimated glomerular filtration rate, *HbA1c* hemoglobin A1c, *LDL-C* low-density lipoprotein cholesterol, *LVH* left ventricular hypertrophy.

### Association of electrocardiographic LVH with incidence of renal function decline

**Figure 2:**
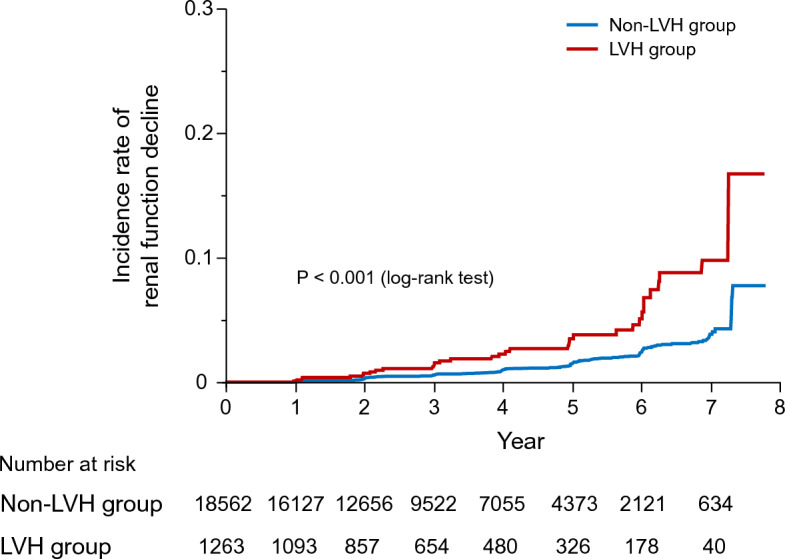
Cumulative incidence curves of renal function decline. Kaplan‒Meier curves show the cumulative incidence of renal function decline in the LVH group and the non-LVH group. This study suggests that electrocardiographic LVH can predict future renal function decline in the general population. *eGFR* estimated glomerular filtration rate, *LVH* left ventricular hypertrophy.

The incidence rates of renal function decline were 0.30 per 100 person-years in the non-LVH group and 0.78 per 100 person-years in the LVH group. Kaplan–Meier curves of the cumulative incidence of renal function decline in the LVH and non-LVH groups are provided in Fig. 2. The incidence of renal function decline was significantly higher in the LVH group than in the non-LVH group (P < 0.001, log-rank test).

In univariate Cox regression analyses, electrocardiographic LVH was associated with future renal function decline (hazard ratio [HR] 2.50, 95% confidence interval [CI] 1.73–3.60, P < 0.001). Higher age, male sex, higher BMI, history of cardiovascular disease, hypertension, higher systolic BP, lower LDL-C, higher HbA1c, higher uric acid, lower eGFR, and urinary protein were also associated with future renal function decline in univariate analyses (Table 2). In the multivariate Cox regression analyses using models 1, 2, or 3, electrocardiographic LVH was significantly associated with future renal function decline regardless of the multivariate model. In model 1, the HR adjusted for age and sex was 1.86 (95% CI 1.29–2.70, P = 0.001). In model 2, the HR adjusted for age, sex, and baseline renal function was 1.83 (95% CI 1.26–2.64, P = 0.001). In model 3, the HR adjusted for age, sex, baseline renal function, BMI, diagnosis of hypertension, systolic BP, and other potential confounders was 1.69 (95% CI 1.14–2.50, P = 0.009; Tables 2 and 3). In sensitivity analyses, electrocardiographic LVH was also associated with the risk for a decrease of ≥ 20% in the eGFR from baseline to < 60 mL/min/1.73 m^2^ (HR 1.43, 95% CI 1.05–1.95, P = 0.022) and a decrease of ≥ 30% in the eGFR from baseline to < 60 mL/min/1.73 m^2^ (HR 1.83, 95% CI 1.10–3.06, P = 0.021) with model 3 (Table 3).

**Table 2:** Unadjusted and adjusted hazard ratios for renal function decline*.

	Unadjusted			Adjusted^#^		
	HR	95% CI	P value	HR	95% CI	P value
Electrocardiographic LVH	2.50	1.73–3.60	< 0.001	1.69	1.14–2.50	0.009
Age (per 1 year increase)	1.08	1.06–1.09	< 0.001	1.05	1.03–1.07	< 0.001
Male	1.42	1.09–1.86	0.009	0.92	0.66–1.29	0.64
BMI (per 1 kg/m^2^ increase)	1.05	1.02–1.09	0.004	1.00	0.95–1.04	0.85
Current smoking	1.24	0.90–1.70	0.19	1.75	1.22–2.50	0.002
History of CVD	3.00	2.07–4.35	< 0.001	1.23	0.82–1.82	0.32
Hypertension	4.18	3.21–5.45	< 0.001	1.79	1.31–2.43	< 0.001
Diabetes mellitus	3.89	2.76–5.48	< 0.001	1.33	0.85–2.07	0.21
Systolic BP (per 1 mmHg increase)	1.03	1.03–1.04	< 0.001	1.01	1.01–1.02	0.001
eGFR (per 1 mL/min/1.73 m^2^ increase)	0.96	0.95–0.97	< 0.001	0.98	0.97–0.99	0.003
Urinary protein positive	4.11	2.75–6.15	< 0.001	1.94	1.25–2.99	0.003
HbA1c (per 1% increase)	1.56	1.41–1.72	< 0.001	1.34	1.14–1.57	< 0.001
LDL-C (per 1 mg/dL increase)	0.99	0.99–1.00	0.005	0.99	0.99–1.00	< 0.001
Uric acid (per 1 mg/dL increase)	1.16	1.06–1.27	0.002	1.04	0.92–1.18	0.53

*Renal function decline was defined as a decrease of ≥ 25% in the eGFR from baseline to < 60 mL/min/1.73 m^2^.

^#^Adjusted hazard ratios were calculated in model 3 that included sex, age, baseline estimated glomerular filtration rate and baseline urinary protein (positive or negative), body mass index, current smoking status, diagnosis of hypertension, diagnosis of diabetes mellitus, history of cardiovascular diseases, systolic blood pressure, low-density lipoprotein cholesterol, uric acid, and hemoglobin A1c.

*BMI* body mass index, *BP* blood pressure, *CI* confidence interval, *CVD* cardiovascular diseases, *eGFR* estimated glomerular filtration rate, *HR* hazard ratio, *LDL-C* low density lipoprotein cholesterol, *LVH* left ventricular hypertrophy.

**Table 3:** Association between electrocardiographic left ventricular hypertrophy and different endpoints of renal function decline.

Outcome	Group	Incidence rate (per 100 py)	Number of events	HR	95% CI	P value
≥ 25% eGFR decline and eGFR < 60 mL/min/1.73 m^2^*	Non-LVH	0.30	189/18,562	Ref.		
	LVH	0.78	34/1263	1.69	1.14–2.50	0.009
Sensitivity analysis with different percentages of eGFR decline						
≥ 20% eGFR decline and eGFR < 60 mL/min/1.73 m^2^	Non-LVH	0.60	371/18,562	Ref.		
	LVH	1.23	53/1263	1.43	1.05–1.95	0.022
≥ 30% eGFR decline and eGFR < 60 mL/min/1.73 m^2^	Non-LVH	0.16	103/18,562	Ref.		
	LVH	0.46	20/1263	1.83	1.10–3.06	0.021

*The results for a ≥ 25% eGFR decline and an eGFR of < 60 mL/min/1.73 m^2^ are provided here to promote a better understanding of Table 2.

*CI* confidence interval, *eGFR* estimated glomerular filtration rate, *HR* hazard ratio, *LVH* left ventricular hypertrophy, *py*. person-years.

**Figure 3:**
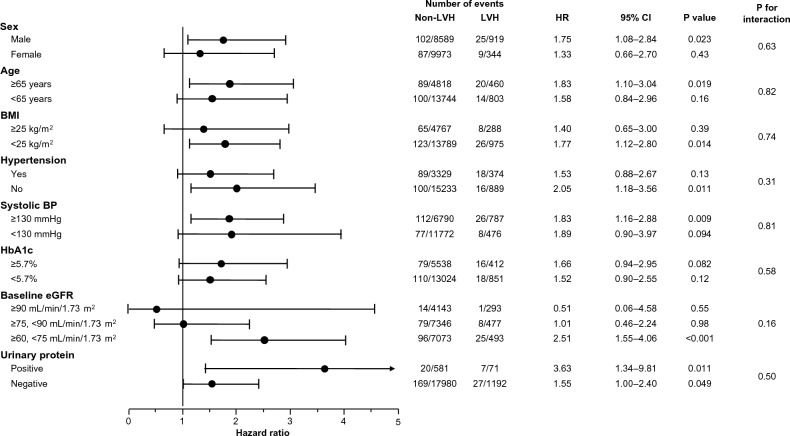
Risk for future renal function decline in different subgroups. Association between electrocardiographic LVH and the risk for future renal function decline in the subgroups stratified by age, sex, body mass index, diagnosed hypertension, systolic blood pressure, hemoglobin A1c, baseline eGFR, and urinary protein. Hazard ratios were calculated by a multivariate Cox proportional hazards model with model 3. *BMI* body mass index, *BP* blood pressure, *CI* confidence interval, *eGFR* estimated glomerular filtration rate, *HbA1c* hemoglobin A1c, *HR* hazard ratio, *LVH* left ventricular hypertrophy.

The results of the subgroup analyses are shown in Fig. 3. The subgroup analysis based on sex, age (≥ 65 vs < 65 years), BMI (≥ 25 vs < 25 kg/m^2^), diagnosis of hypertension, systolic BP (≥ 130 vs < 130 mmHg), HbA1c (≥ 5.7 vs < 5.7%), eGFR (≥ 90 vs ≥ 75, < 90 vs ≥ 60, < 75 mL/min/1.73 m^2^), and urinary protein (positive vs negative) did not find a significant interaction between electrocardiographic LVH and these factors for the association with future renal function decline.

## Discussion

In this study, the incidence of renal function decline was higher in the LVH group than in the non-LVH group in the general population. After adjustment for potential confounding factors, electrocardiographic LVH was significantly associated with future renal function decline. Subgroup analyses considered the potential effects of sex, age, BMI, diagnosis of hypertension, systolic BP, HbA1c, and urinary protein on electrocardiographic LVH or renal function decline (Fig. 3). These analyses revealed that the main result of our study was basically consistent across subgroups. In particular, the subgroup analysis based on the presence or absence of a hypertension diagnosis and based on systolic BP indicated that electrocardiographic LVH was similarly associated with future renal function decline between the participants with and without the diagnosis of hypertension and between those with and without elevated BP of ≥ 130 mmHg, which can extend the finding from a previous study reporting that electrocardiographic LVH was associated with the risk of progression of CKD in patients with hypertension^14^. The subgroup analyses, together with the multivariate analyses, potentially confirm the association between electrocardiographic LVH and future renal function decline in the general population.

It should be noted that the primary renal outcome in this study was defined as a decrease of ≥ 25% in the eGFR from baseline to < 60 mL/min/1.73 m^2^. This definition differs from commonly employed robust surrogate outcomes, such as a 40% decrease in eGFR. Our study aimed to elucidate the clinical usefulness of electrocardiographic LVH for early identification of high-risk individuals for CKD within the general population, i.e., apparently healthy individuals who have not yet progressed to CKD stage G2, in the setting of health checkups. It is common practice to categorize individuals with an eGFR < 60 mL/min/1.73 m^2^ as already having CKD and warranting referral to a physician or nephrologist. Therefore, according to the aim of this study, we excluded the participants with eGFR < 60 mL/min/1.73 m^2^ at baseline. It is important to acknowledge that, owing to this study design, the primary renal outcome of our study may not reflect hard outcomes such as end-stage renal failure or death due to renal failure. While greater declines in eGFR, such as a 40% eGFR decline, might better reflect hard renal outcomes, our study, in which the majority of participants did not experience a significant decline in renal function, lacked sufficient statistical power to analyze more robust surrogate outcomes.

Electrocardiographic LVH is a common electrocardiographic abnormality^16^ and is associated with older age, smoking, higher BMI, albuminuria, and hypertension^16,17^. In this study, the LVH group was older than the non-LVH group (Table 1). Additionally, male participants, current smokers, individuals with a history of cardiovascular disease, and those with hypertension were more frequently observed in the LVH group. Systolic BP at the baseline visit was also higher in the LVH group than in the non-LVH group. These factors have been reported to be associated with renal function decline^18–20^. Although the higher incidence of renal function decline in the LVH group may be partly due to these participants’ characteristics, the multivariate Cox regression analysis revealed a significant association between electrocardiographic LVH and renal function decline after adjusting for these potential confounders (Table 2). Therefore, while occult heart disease that was not counted as a cardiovascular event, such as asymptomatic chronic heart failure, may not have been reflected in the analyses, the presence of electrocardiographic LVH itself appeared to be associated with progression of renal dysfunction independently of participant characteristics that may be related to renal function decline.

The specificity of several electrocardiographic LVH criteria, including the Minnesota code used to detect increased left ventricular (LV) mass, was high, whereas the sensitivity was low^21,22^. Thus, most individuals with electrocardiographic LVH should be accompanied by truly increased LV mass. A cross-sectional analysis showed that increased LV mass defined by echocardiogram was associated with reduced eGFR and an increased urine albumin/creatinine ratio^23^. Indeed, a study analyzing autopsy specimens showed that a lower eGFR was associated with increased LV wall thickness, greater cardiac cell size, and area of LV fibrosis^12^. In longitudinal observations, increased LV mass by echocardiogram was also associated with the incidence of newly developed CKD or renal function decline in patients with diabetes, patients with CKD, and the general population^24–26^. These studies have indicated the underlying pathological relationship between the heart and the kidney. The renin–angiotensin system is well established to be associated with myocardial hypertrophy^27^. In addition to the renin–angiotensin system, overactive sympathetic nervous system, inflammation, and oxidative stress are associated with increased LV mass^28–30^. All these factors have been found to cause and exacerbate pathophysiological changes in CKD^31,32^. Thus, individuals with increased LV mass may be exposed to these risk factors for renal damage and predisposed to future renal function decline even though their renal function has not diminished.

### Strengths and limitations

One of the strengths of this study lies in the number of participants in the dataset derived from health checkups, totaling more than 19,000 individuals from the general population. Furthermore, it is an advantage that electrocardiography is often performed not only in daily clinical practice for patients with diseases such as hypertension, diabetes mellitus, or dyslipidemia, but also in health checkups for those who have not seen a clinician. In addition, the interobserver difference appears to be lower in the electrocardiogram than in the echocardiogram. Our study suggests that electrocardiogram, which is a common examination, may be useful to detect individuals at high risk for renal dysfunction who should be given earlier therapeutic intervention or cautious follow-up in health checkups and daily practices. Conversely, this study has several limitations. First, this study used the criteria for electrocardiographic LVH of the Minnesota code, which is commonly used in health checkups, at least in Japan. Various criteria for electrocardiographic LVH have been proposed^21,22^. Thus, our results might be different when similar analyses are performed with other criteria. In addition, we could not perform quantitative assessments, such as of R or S wave amplitude or QRS duration. Second, although the majority of individuals underwent annual health checkups, during which eGFR measurements were performed each year, some individuals did not have health checkups annually. Therefore, not all eGFR measurements were at a fixed interval of 1 year. Third, the presence of a hypertension diagnosis was based on a questionnaire response confirmed by a public health nurse. Participants who were not properly informed or aware of their hypertension diagnosis, as well as those with masked hypertension, would have been included in the group of participants without a diagnosis of hypertension. Fourth, our dataset did not include medication data, and we could not adjust for the use of drugs with renal protective effects, such as angiotensin-converting enzyme inhibitors/angiotensin II receptor blockers or sodium-glucose transporter 2 inhibitors^33,34^. Finally, we could not analyze the parameters of echocardiogram or cardiac magnetic resonance imaging, which can present LV mass. Thus, it remains unclear whether the association between electrocardiographic LVH and future renal function decline is still significant after adjustment for LV mass. It would be beneficial to distinguish the effect of electrocardiographic LVH from the effect of increased LV mass. Further studies are needed to clarify this issue.

## Conclusions

Electrocardiographic LVH was associated with a future decline in renal function in the general population. This association was consistent regardless of sex, age, BMI, diagnosis of hypertension, systolic BP, HbA1c, and urinary protein among participants. Electrocardiogram may be useful to detect individuals at high risk for future renal function decline in the setting of health checkups of the general population.

## Methods

### Data source

We acquired data from JA Ehime Kouseiren Checkup Center. This study was approved by the local ethics committee at Kyushu University Hospital (Approval no. 22181-00) and Ehime University (Approval no. 1912011). This study complied with the Declaration of Helsinki. Informed consent was obtained through opt-out on the website in this retrospective and noninterventional study.

### Study population

This retrospective cohort study used population-based health checkup data. A dataset derived from the annual health screening program performed by JA Ehime Kouseiren Checkup Center from 2010 to 2019 was used. All the study participants received health checkups at least twice between 2010 and 2019. Individuals were followed from their first (baseline) visit between 2010 and 2018 to their last visit between 2011 and 2019. Individuals lacking baseline eGFR or electrocardiography data were excluded from the study. Individuals with baseline eGFR < 60 mL/min/1.73 m^2^ were also excluded, because individuals with eGFR < 60 mL/min/1.73 m^2^ might already have CKD at baseline. Individuals lacking eGFR during the follow-up period were also excluded.

During health checkups, all participants were asked to fill out questionnaires asking about their past medical history, medications, smoking, and daily practice. Public health nurses confirmed the answers to the questionnaires to the participants in person. Participants were subjected to anthropometric measurements, BP measurements, blood tests, urine dipstick tests, and physical examinations by physicians.

### Data collection

LVH was defined according to Minnesota codes 3-1 (R amplitude > 2.6 mV at V5 or V6 leads, R amplitude > 2.0 mV at I, II, III, or aVF leads, or R amplitude > 1.2 mV at aVL lead) and 3-3 (R amplitude > 1.5 mV and ≤ 2.0 mV at I lead, or sum of R amplitude at V5 or V6 lead and S amplitude at V1 leads > 3.5 mV)^35^. The Minnesota code was introduced to objectively confirm electrocardiographic findings and is commonly used in the setting of health checkups. The diagnosis of LVH was based on an automatic electrocardiographic diagnosis system and was subsequently confirmed by physicians at JA Ehime Kouseiren Checkup Center.

Histories of cardiovascular diseases, cerebrovascular diseases, and comorbidities such as hypertension, dyslipidemia, or diabetes mellitus were based on questionnaire responses. BP was measured twice in the sitting position in all participants, and the mean value was used. Blood samples were analyzed in the laboratory of JA Ehime Kouseiren Checkup Center. Serum creatinine concentration was measured, and the eGFR was calculated in each annual visit using the following criteria: eGFR (mL/min/1.73 m^2^) = 194 × (serum creatinine concentration [mg/dL])^−1.094^ × age^−0.287^ (for males), or (eGFR for males) × 0.739 (for females), which are revised equations for Japanese individuals^36^. Urine dipstick results were judged by the medical staff and recorded as (−), (±), (1+), (2+), and (3+). Urinary protein was regarded as positive when the urine dipstick result was ≥ 1+, which corresponds to a urinary protein level of ≥ 30 mg/dL^37^. The dataset of this study was obtained from annual health checkups, and the frequency of testing for each parameter, including eGFR, was basically once a year.

### Outcomes

The primary outcome of this study was renal function decline, which was defined as a decrease of ≥ 25% in the eGFR from baseline to < 60 mL/min/1.73 m^2^. As sensitivity analyses, we also analyzed the association for (1) a decrease of ≥ 20% in the eGFR from baseline to < 60 mL/min/1.73 m^2^ and (2) a decrease of ≥ 30% in the eGFR from baseline to < 60 mL/min/1.73 m^2^.

### Statistical analysis

Participants were divided into two groups based on baseline electrocardiographic findings: individuals with and without electrocardiographic LVH (LVH group and non-LVH group, respectively). Continuous variables are expressed as the mean and standard deviation and were analyzed using the *t* test unless otherwise noted. All categorical variables were expressed as raw numbers and percentages and were compared using the chi-square test.

Kaplan–Meier cumulative incidence curves were compared by the log-rank test. Multivariate Cox proportional hazards models were used to calculate the hazard ratios for the incidence of renal function decline. For adjusting potential confounding factors, we developed three models. Model 1 included sex and age. Model 2 additionally included baseline eGFR and baseline urinary protein (positive or negative). Model 3 further included body mass index (BMI), current smoking status, diagnosis of hypertension, diagnosis of diabetes mellitus, history of cardiovascular disease including heart disease or stroke, systolic BP, LDL-C, uric acid, and HbA1c at baseline. To determine whether the association between electrocardiographic LVH and renal function decline was affected by sex, age, BMI, diagnosis of hypertension, systolic BP, HbA1c, eGFR, and urinary protein, subgroup analyses were additionally performed using model 3. A two-sided P value < 0.05 indicated statistical significance. All the analyses were conducted with SAS 9.4 (SAS Institute Inc., Cary, NC, USA).

## Data Availability

The datasets generated during and/or analyzed during the current study are not publicly available due to ethical restrictions but are available from the corresponding author on reasonable request.
